# A Population-Based Study of Sex Differences in Cardiovascular Disease Mortality Among Adults with Ocular Cancer in the United States, 2000–2021

**DOI:** 10.3390/curroncol32080447

**Published:** 2025-08-08

**Authors:** Duke Appiah, Abdulkader Almosa, Eli Heath, Noah De La Cruz, Obadeh Shabaneh

**Affiliations:** 1 Julia Jones Matthews School of Population and Public Health, Texas Tech University Health Sciences Center, Lubbock, TX 79430, USA; 2 College of Arts and Sciences, Texas Tech University, Lubbock, TX 79409, USA; aalmosa@ttu.edu; 3 Department of Chemical Engineering, Texas Tech University, Lubbock, TX 79409, USA; eliheath@ttu.edu; 4 Department of Internal Medicine, Texas Tech University Health Sciences Center, Amarillo, TX 79106, USA; noah.de-la-cruz@ttuhsc.edu; 5 School of Medicine, St. George’s University, University Centre, St. Georges FZ818, Grenada; oshabane@sgu.edu

**Keywords:** ocular cancer, cardiovascular disease, sex differences, epidemiology, mortality

## Abstract

Little is known about the manifestation of cardiovascular diseases (CVD) among individuals with ocular cancer (OC), a population for whom reports on sex-based differences in survival remain inconsistent. We evaluated the occurrence of CVD mortality after the diagnosis of OC in the United States. We used data from 11,460 adults diagnosed with OC from 2000 to 2021 who were ≥18 years and were enrolled in the Surveillance, Epidemiology, and End Results program. We used competing risk models to estimate hazard ratios (HR) and 95% confidence intervals (CI). About 55% of adults were male, with uveal melanoma being the most common OC (72.1%). During a median follow-up of 5.4 years, 4561 deaths occurred, with 15% attributable to CVD. In models adjusted for sociodemographic and clinico-pathophysiological factors, male adults had elevated risk for CVD mortality (HR: 1.54, 95%CI: 1.31–1.81). The sex difference in CVD mortality was more prominent for adults diagnosed with OC before 65 years of age (HR: 2.15; 95%CI: 1.48–3.11). These associations remained largely unchanged in propensity score analysis. In this study of adults with OC, CVD deaths were higher among young and middle-aged males. Implementation of optimal cardiovascular health interventions after diagnosis of OC, especially among men, holds promise in enhancing survival in this population.

## Simple Summary

Emerging evidence suggests an association between ocular and cardiovascular health. However, little is known about the relationship between ocular cancer and cardiovascular disease. In this study of adults in the United States with ocular cancer, cardiovascular disease-related deaths were higher among male adults. Young and middle-aged male adults with ocular cancer had the highest burden of cardiovascular disease-related deaths.

## 1. Introduction

Ocular cancer (OC) is a rare and diverse malignancy that accounts for approximately 0.2% of all cancer cases, and less than 0.1% of cancer-related deaths worldwide [1,2]. In the United States, about 3320 new cases and 560 deaths due to OC are reported each year [3]. In contrast to the minimal annual increase of 0.3% in the overall incidence of OC during the past 20 years, there has been a corresponding 1.8% increase in mortality among individuals with OC over the same time period, with the annual percent change significantly increasing threefold (5.3%) from 2018 to 2022 [4].

Despite extensive research on OC, the underlying causes are not well established in adults. While primary OC may affect either intraocular or extraocular tissues, with the most common primary malignancies among adults being ocular melanoma, secondary intraocular malignancies may occur from cancers that metastasize from other sites, such as the breast or lungs [1,5]. The prognosis of OC is relatively favorable, with 5- and 10-year overall survival of 75.3% and 59.8% [6]. However, the malignancies affecting the uvea have a significant impact on the quality of life of individuals due to vision loss [1].

On the one hand, sex-based differences in tumor characteristics and location, as well as metastatic behavior and treatment, have been widely reported, with men often having earlier and higher incidence and more frequent metastases than women [7,8,9,10,11,12]. On the other hand, evidence for sex-based survival remains equivocal [7,8,13,14,15,16]. Most studies on sex differences in survival after OC diagnosis have largely focused on uveal melanoma, the most common primary intraocular tumor in adults in the United States [7,8,13,14]. Thus, further investigations of sex-based differences in survival outcomes of populations, including other histologic subtypes of OC, are needed to inform the clinical care of individuals with ocular tumors.

In the face of decreasing cancer deaths in the United States [3], non-cancer deaths, namely those attributed to cardiovascular disease (CVD), among individuals with cancer are currently on the rise [17]. Accordingly, a recent study reported that some cancer survivors are at an elevated risk of premature CVD mortality compared to the general population [18]. Accumulating evidence suggests a significant overlap between ocular and cardiovascular health [19,20,21,22,23]. However, little is known about the manifestation of CVD among adults with ocular malignancies. Therefore, the aim of this study was to evaluate the risk of CVD mortality following diagnosis of OC in a large population-based cohort in the United States.

## 2. Materials and Methods

### 2.1. Study Population

**Figure 1:**
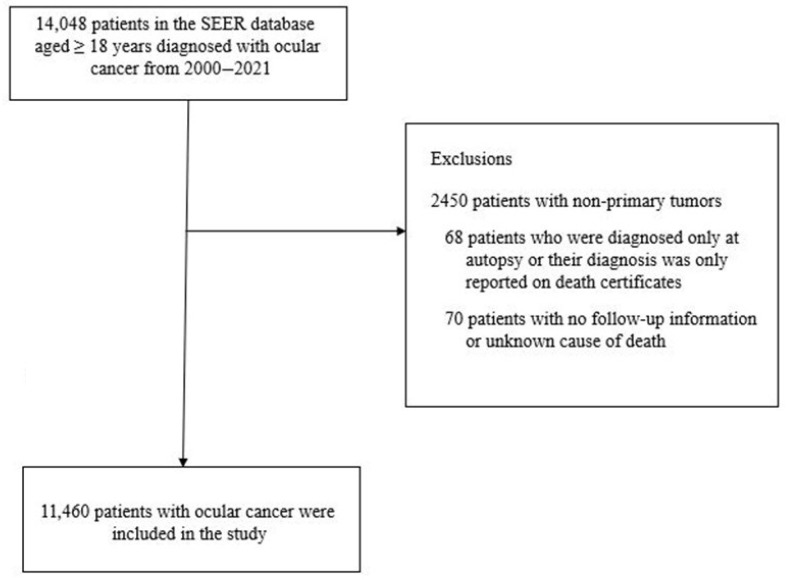
Flow chart of sample selection for the current study, SEER 2000–2021.

The current study used data from the Surveillance, Epidemiology, and End Results (SEER) program. This program includes 21 cancer registries, which cover 48% of the population of the United States [24]. The SEER program database was queried for adults with OC who were aged ≥18 years and were diagnosed from 2000 to 2021 using International Classification of Diseases for Oncology, Third Edition (ICD-O-3) topographical codes (C69.0–69.9). From the 14,048 adults who met the above-mentioned eligibility criteria, the following exclusions were made: individuals with non-primary tumors (n = 2450); those who were diagnosed only at autopsy or had their diagnosis only reported on death certificates (n = 68); and those with no follow-up information or unknown cause of death (n = 70). These exclusions yielded an analytic sample of 11,460 (Figure 1). For the current study, approval from an institutional review board was not required since the data from the SEER program is de-identified and publicly available.

### 2.2. Definition of Study Variables

Sex—the exposure variable of interest for this current study—was classified as male or female, according to SEER-defined sex categories. Other sociodemographic and socioeconomic information obtained from the database includes year of diagnosis, age at diagnosis, race and ethnicity, geographic region, rural or urban location of residence, marital status, and county-level annual median household income. Clinical and pathophysiological factors evaluated were the primary site of tumor, histology, tumor stage, laterality, mode of diagnosis, time from diagnosis to treatment, initial cancer therapy, cause of death, and survival time. Because of limited information available for tumor size and grade for adults with OC, these factors were not considered in the current study. Histologic features and behavior of ocular tumors were determined using ICD-O-3 morphology codes 8720 to 8790/3 for melanoma and 8050 to 8089/3 for squamous cell neoplasms. Melanomas were further classified using ICD-O-3 topographical codes C69.3 (choroid) and C69.4 (ciliary body and iris) for uveal melanoma and C69.0 (conjunctiva) for conjunctival melanoma [25]. Due to very small sample sizes of retinoblastoma (9510 to 9514/3) in the analytic sample, it was classified together with other histologic subtypes as “Other histologic subtypes”. Information on the underlying cause of death was obtained using ICD-10 codes. The following codes were used to define CVD mortality: I00–I09, I11, I13, I20–I51 (diseases of heart), I10–115 (hypertensive heart disease), I60–I69 (cerebrovascular diseases), I70 (atherosclerosis), I71 (aortic aneurysm and dissection) and I72–I78 (other diseases of arteries, arterioles, capillaries [26].

### 2.3. Statistical Analysis

Characteristics of adults with OC were described and compared by sex using a chi-square test. It is possible that the age distribution of patients with OC may be different from the general population, thus influencing a higher CVD mortality in the latter. Therefore, age-standardized mortality ratios were calculated to compare CVD mortality among patients with OC to CVD mortality in the U.S. general population.

To estimate the association of sex with mortality outcomes among patients with OC, we employed competing risk analyses (cause-specific hazard models). For CVD mortality, all deaths not due to CVD were regarded as competing risk events. The following variables were included in the multivariable model as potential confounders based on a review of the literature: age at diagnosis, year of diagnosis, race and ethnicity, marital status, geographic region, location, income, histology, tumor site, tumor stage, laterality, mode of diagnosis, time from diagnosis to treatment, and initial cancer therapy. Owing to reported sex differences in clinical presentation and prognosis of OC [7], formal interaction tests between sex and age, race and ethnicity, tumor site, tumor stage, and treatment modalities were conducted using the Wald test and reported whenever statistically significant. The cumulative sums of martingale residuals with a Kolmogorov-type supremum test were used to test the proportional hazards assumption. When missing data were present, they were coded as unknown. Although all these analyses were pre-specified, adjusted *p* values were calculated to control the false discovery rate using the Benjamini–Hochberg adaptive step-up procedure.

To control for the effect of measured confounding factors and to minimize potential sex bias related to the diagnosis and treatment of OC influencing the observed outcome, we used a logistic regression model to estimate propensity scores. Factors included in the logistic model to match males and females (1:1 match) were age at diagnosis, year of diagnosis, race and ethnicity, marital status, geographic region, income, tumor site, tumor stage, laterality, mode of diagnosis, time from diagnosis to treatment, and initial cancer therapy. The nearest-neighbor greedy matching algorithm was employed. For this algorithm, we set the calipers at 0.25 of the standard deviation of the logit of the propensity score. Negligible differences in the proportion of covariates between the matched pairs were defined as an absolute standardized difference (ASD) of less than 0.2 [27]. We repeated all previously estimated hazard ratios using the propensity score matched samples. The SEER*Stat version 8.4.4 software (Information Management Systems, Rockville, MD, USA) was used to extract data from the SEER program and calculate standardized mortality ratios, while the SAS software version 9.4 (SAS Institute, Inc., Cary, NC, USA) was used for statistical analyses, with statistical significance set at a *p* value of less than 0.05.

### 2.4. Verification Analysis

To evaluate the robustness of the findings of the current study, the inferential analyses were repeated using the data from the Texas Cancer Registry (TCR), which consists of 2832 adults diagnosed with OC from 1995 to 2019 who met the aforementioned inclusion criteria listed for the current study. While TCR data is included in SEER 21, the data used for the main analysis were from SEER 17, which did not include data from Texas; thus, these two databases are mutually exclusive [24]. The TCR used data coding procedures and variable definitions similar to SEER. For the multivariable analyses, the same covariates used in the main analyses were included in the models for the verification analyses with the exception of three variables. Region was not included since the regions of Texas do not correspond with the regions for SEER, while mode of diagnosis and time from diagnosis to treatment were not included in the publicly available TCR database. However, the TCR database had more specific sociodemographic variables like health insurance status and neighborhood poverty, which were included in the verification analysis.

## 3. Results

The mean age of adults with OC was 61.5 ± 14.9 years, with approximately 55% of them being male adults. The racial and ethnic distribution of adults with OC was as follows: non-Hispanic White, 85.5%; non-Hispanic Black, 1.7%; Hispanic, 8.3%; and Asian or Pacific Islander, 2.4%. More than half of adults with OC (55.3%) lived in counties with median household incomes of less than USD 75,000. With regard to clinical factors, uveal melanoma was the most prevalent histologic subtype (72.1%), with the choroid being the most common primary site (63.2%). For comparison, retinoblastomas were very rare in this population (0.05%). At the time of diagnosis, two-thirds (65.2%) of adults had stage I (localized) tumors. Characteristics of participants by sex are presented in Table 1. Compared to female adults, a greater proportion of male adults were diagnosed with OC at younger ages, were married, had positive histology tests for confirmation of cancer diagnosis, often had tumors affecting the conjunctiva, received treatment in less than a month of diagnosis, and underwent surgery or used chemotherapy as an initial treatment regimen. There were no sex differences in race and ethnicity, median household income, tumor stage, and tumor laterality at the time of diagnosis.

**Figure 2:**
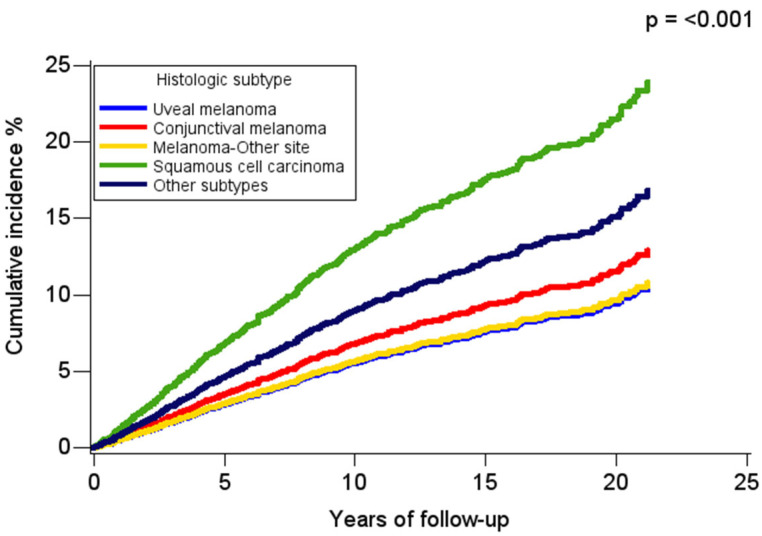
Cumulative incidence curves for cardiovascular disease-related deaths among adults with ocular cancer by histologic subtypes, SEER 2000–2021. The *p* value represents Gray’s test for equality of cumulative incidence functions.

During a median follow-up of 5.4 (interquartile range: 2.3–10.3) years, 4561 deaths occurred, with 21% attributable to OC and 15% attributable to CVD, which represented almost half (42%) of all non-cancer causes of death. Comparing the frequency of CVD deaths between adults with OC to the general population, it was observed that male adults with OC who were <65 years of age had higher excess CVD mortality (standardized mortality ratio: 1.39; CI: 1.06–1.78), while female adults with OC who were >65 years had lower excess risk than the general population of women (standardized mortality ratio: 0.87; CI: 0.77–0.99). In time-to-event analysis, the cumulative incidence of CVD deaths varied by histologic subtype, with CVD mortality highest for conjunctival squamous cell carcinoma and lowest for uveal melanoma (Figure 2).

**Figure 3:**
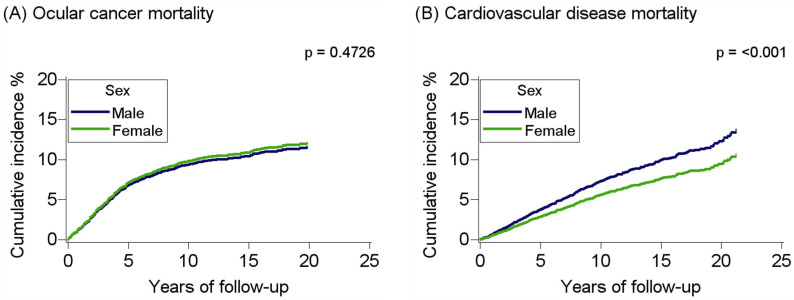
Cumulative incidence curves for ocular cancer mortality (**A**) and cardiovascular disease mortality (**B**) among adults with ocular cancer by sex, SEER 2000–2021. The *p* values represent Gray’s test for equality of cumulative incidence functions.

With regard to sex, the cumulative incidence of CVD deaths was higher among males compared to females with OC (Figure 3). In age-adjusted models, male adults had an elevated risk for CVD mortality than female adults (HR: 1.41; 95%CI: 1.21–1.64), with the risk increasing in fully adjusted models (HR: 1.54; 95%CI: 1.31–1.81, *p* < 0.001; *p* (adjusted) < 0.001) (Table 2). Male adults with OC also had elevated risk for all-cause mortality; however, the estimate was lower in comparison to those for CVD mortality (HR: 1.20; 1.13–1.27, *p* < 0.001; *p* (adjusted) < 0.001). No sex differences were observed for OC mortality (HR: 1.02; 0.89–1.16, *p* = 0.7738).

**Figure 4:**
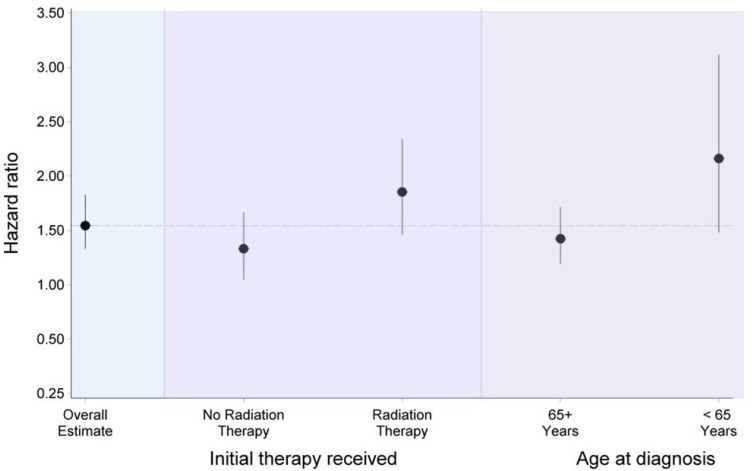
Sex difference (male vs. female) in cardiovascular disease mortality among adults with ocular cancer by initial therapy received (radiation) and age at diagnosis, SEER 2000–2021. The black dots represent hazard ratios and the vertical black lines passing through the black dots are the corresponding 95% confidence intervals for the estimates for males compared to females, with females being the referent group.

There was a significant interaction (*p* = 0.0376; *p* (adjusted) = 0.0454) between sex and age, with the sex difference in CVD mortality greater for adults diagnosed with OC before 65 years (HR: 2.15; 95%CI: 1.48–3.11) compared to those diagnosed with OC at or after age 65 years (HR: 1.41; 95%CI: 1.17–1.70). Additionally, the sex-difference in CVD deaths also varied by initial treatment received (*p* = 0.0389; *p* (adjusted) = 0.0454), with adults receiving radiation therapy having greater risk than those who did not receive radiation therapy (HR: 1.84, 95%CI: 1.45–2.33 vs. HR: 1.31, 95%CI: 1.04–1.65) (Figure 4). There was no significant interaction between sex and race and ethnicity, tumor site, and tumor stage on the risk of CVD mortality. Propensity score matching yielded 8980 (male: 4490, female: 4490) adults who were well balanced on baseline characteristics (Table 3). The hazard ratios for sex differences in CVD mortality, as well as ocular cancer mortality and all-cause mortality, were all very similar to those obtained from models without propensity score adjustments (Table 2).

Participants in TCR shared some similar characteristics with participants in the main analysis. For instance, the mean age of adults with OC in the TCR was 61.2 ± 15.6 years, with 53% of them being males. The racial and ethnic distribution of adults with OC was as follows: non-Hispanic White, 84.4%; non-Hispanic Black, 2.0%; and Hispanic, 12.0%. Uveal melanoma was the most prevalent histologic subtype (68.3%), with the choroid being the most common primary site (56.7%). Characteristics of participants by sex in TCR are presented in Table 4. During a median follow-up of 4.8 (interquartile range: 2.1–10.5) years, 1302 deaths occurred, with 19.5% attributable to CVD. In both age-adjusted and multivariable adjusted models, males have a higher risk for all-cause (HR = 1.14, 95%CI: 1.02–1.28) and CVD mortality (HR = 1.34, 95%CI: 1.03–1.76) (Table 5). Similarly to the main analysis, there was a significant interaction between sex and age (*p* = 0.0130), with males < 65 years being at the highest risk for CVD death (HR = 2.24, 95%CI: 1.13–4.44). However, the interaction between sex and radiation on CVD mortality was not statistically significant.

## 4. Discussion

The findings from this population-based study showed that after the diagnosis of OC, male adults have a higher risk of CVD death compared to female adults. This sex difference was more prominent for adults diagnosed with OC before age 65 years compared to those who were diagnosed with OC at 65 years or older. These findings were consistent across an external database, confirming the robustness of the results of the current study. To our knowledge, the current study is the first to comprehensively evaluate CVD mortality among individuals diagnosed with ocular malignancies.

While a decreasing trend in cancer mortality has been observed in the United States [3], non-cancer deaths, mainly due to CVD, are gradually increasing among cancer survivors [17,18]. Owing to advancements in therapeutics, survival after diagnosis for some cancers like OC has improved, leading to the overlap of cancer and other chronic diseases, including CVD [18,28]. Although limited, there is evidence supporting an inverse relationship between ocular and cardiovascular health [19,20,21,22], with shared pathophysiological pathways such as inflammation and metabolic dysfunction often implicated. For instance, metabolic conditions like diabetes and hypertension are associated with a greater proportion of ocular conditions that are unrelated to refractive errors [29]. These cardiometabolic conditions often drive microvascular changes in the ocular vasculature, such as narrow retinal arterial diameters and wide retinal venular diameters through processes such as endothelial dysfunction and inflammation [29,30]. Chronic hyperglycemia also exerts ocular tissue-damaging effects via oxidative stress, which drives the heightened production of reactive oxygen species [29]. High levels of intracellular reactive oxygen species cause irreversible damage to cells in the eye through epigenetic changes such as DNA methylation and histone modifications [29]. Chronic low-grade inflammation affects the ocular vasculature through capillary occlusion and hypoxia [29], while also leading to the overexpression of vascular endothelial growth factor, which influences ocular angiogenesis [31]. The novel findings of this current study contribute to this growing body of evidence in at least two ways. First, they show that there is an overlap between CVD and ocular malignancies. Second, they show that among this observed overlap, there is a sex difference, which happens to be prominent in young and middle-aged adults.

The observed relationship between CVD and OC in this study may, in part, be explained by shared risk factors between these two conditions. The association of metabolic conditions like obesity and diabetes with both CVD and ocular malignancies like ocular melanoma suggests a shared biological process between these two diseases [32,33]; although an obesity paradox has been described for uveal melanoma [34]. For instance, abnormal glucose metabolism and insulin resistance, which are associated with the occurrence of CVD, also influence the development and progression of intraocular cancers through several pathways [35]. These include creating an adiponectin-deficient environment, which limits the binding of Adipor1, the receptor of adiponectin, to uveal melanoma cells to exert its antitumor functions [35,36]. Furthermore, insulin resistance promotes overproduction of insulin-like growth factor-1, which, upon binding to IGF-1R, activates mitogen-activated protein kinase and phosphatidylinositol 3 kinase signaling pathways that enhance the genesis and metastasis of ocular melanoma tumors [35]. Other shared biological pathways linking CVD and OC include hypoxia and inflammation, where cytokines and factors like hypoxia inducible factor 1 alpha, interleukin 6, Interleukin 8, and monocyte chemotactic protein 1 have been implicated in the prognosis and progression of both ocular tumors and CVD [33,37]. A few studies suggest that OC metastases to the heart via a hematogenous route, resulting in fatal outcomes in less than a year [38]. Accordingly, it was reported that almost a quarter of individuals who died of disseminated uveal melanoma were observed to have had cardiac metastases at autopsy [39].

Although not conclusive, there are several factors that may potentially explain the high risk of CVD mortality in males compared to female adults with OC in the current study. It is possible that the observed sex differences may reflect the sex difference in CVD risk profiles, where men often tend to have a greater burden than women; a pattern that is also partly influenced by differences in behavior/lifestyles between men and women [40]. Accordingly, several reports highlight a higher incidence of OC and mortality among men [7,8,13,15]. The higher burden of CVD risk factors in men often leads to them having a more inflammatory microenvironment than women [41]. Furthermore, women have accelerated resolution of inflammation compared to men, as well as lower systemic inflammation-induced endothelial dysfunction [42]. In analyzing the differential gene expression profiles in uveal melanoma tumors from men and women, Liu-Smith et al. [41] reported higher overexpression of genes related to immune function in men than in women. This suggests that CVD risk factors may provoke more immune responses to curtail inflammation in men than women with OC [41], which, in turn, may lead to a higher incidence and mortality from CVD among men with OC compared to women with OC. With CVD risk factors not assessed at baseline in the current study, further studies are warranted to understand the underlying mechanism for the sex difference in CVD mortality among individuals with OC.

Another novel finding of our study is that the sex difference in CVD mortality was greater for young and middle-aged adults diagnosed with OC compared to elderly adults with ocular malignancies. This age-dependent sex difference in CVD mortality among adults with OC may not only reflect the behavior difference in men and women at different ages that predispose them to these two diseases, but also certain intrinsic factors that potentially mediate inflammation and immune response [7,41]. In this respect, it would be assumed that changes in sex hormones, which are age-dependent, may explain the reported association, as receptors for these hormones exist in the ocular system. Female sex hormones that affect physiological conditions like pregnancy and menopause are reported to favorably influence cellular activities, which include immune responses and oxidative regulation [41,43]. However, evidence of a hormonal mechanism showing direct involvement of endogenous female sex hormones like estrogen or the expression of their receptors in the etiology of both CVD and ocular malignancies remains debated [44,45,46]. Although testosterone supplementation is associated with retinal artery occlusion [47], a condition reported to subsequently lead to the development of eye, brain, or central nervous system tumors [48], evidence for a relationship between endogenous androgens and ocular cancer is currently not available.

The treatment-related cardio-toxic side effects of radiation therapy have also been reported to contribute to the development and mortality from CVD in individuals with non-ocular cancers. With a greater proportion of female adults having received radiation therapy in the current study, our finding of males having a higher risk of CVD mortality among individuals who received radiation is paradoxical, as women with cancer are often reported to have more radiation-induced cardiovascular events [49]. Among the few studies that have evaluated sex-related differences in the effectiveness of radiotherapy, female patients with choroidal melanoma were reported to have a lower all-cause mortality rate, although no sex difference was found with choroidal melanoma-specific mortality [50]. This observation has also been observed among other non-ocular cancers [51]. We speculate that a greater burden of CVD risk in men who received radiation therapy may be a possible reason to explain our finding, as such preexisting CVD risk factors elevate the risk of developing radiation cardiotoxicity [49]. In support of this, there is some evidence supporting continuous exposure to radiation, even at lower doses or low dose rate, leading to cataract [52,53], which has been reported to be associated with an elevated risk for CVD mortality [54].

Another possible explanation for the sex difference in CVD deaths varying by the receipt of radiation therapy may be that male patients with OC are often diagnosed at later stages, which manifests in them having larger tumors at the time of diagnosis than female patients with OC [9]. Among individuals with cataract, men are less likely to seek treatment and often present with a greater CVD risk burden than women [55]. Indications for radiation therapy have expanded over the years to include large ocular tumor cells, and the effect of this therapy in the presence of comorbidities such as hypertension and diabetes often results in radiation-induced complications like cataract, glaucoma, and retinopathy within 6 months to 3 years, and these conditions are also reported to be associated with CVD [56,57,58,59]. Toxic tumor syndrome, which usually occurs with irradiation of large tumors, results in the release of inflammatory cytokines by the necrotizing tumor in addition to the proliferation of vascular endothelial growth factor, leading to chronic inflammation [56,57]. However, in the current study, no sex differences were found in the OC stage and time to treatment. It is important to note that information on specific radiation regimens, duration of treatment, and dosage were not available in the SEER database. Also, radiation cardiotoxicity often occurs 10 or more years after treatment [49]. In the current study, less than a quarter of adults had 10 or more years of follow-up. Taken together, the finding of radiation moderating the sex difference in the risk of CVD mortality among adults with OC should be interpreted with caution until confirmed by other studies with information on preexisting conditions at the time of cancer therapy and the development of health conditions before the onset of CVD mortality.

Limitations of the current study are as follows: There were no data on CVD risk factors or data on other noncancer comorbid diseases at the time of cancer diagnosis. Also, data on lifestyle factors, genetic predisposition, and healthcare access were not available. With sex differences often reported in the prevalence of certain CVD risk factors like smoking, body mass index, and hypertension, these unmeasured confounders may result in residual confounding. In light of that, we employed methods like propensity score matching that have good control of confounding factors. Regardless, the possibility of residual confounding affecting the results of the current study cannot be entirely ruled out. Detailed treatment modalities are not available in the SEER program database. The effect of any temporal changes in diagnostic criteria and treatment protocols occurring over the 21-year duration of this study cannot be directly estimated. Despite the SEER program reporting good validity for the ascertainment of cause-of-death information [60], the potential for misclassification of CVD deaths among individuals with cancer based on the use of death certificates, as well as the possibility of differential monitoring intensity between sexes, cannot be entirely ruled out. Finally, findings of this study may or may not be generalizable to all patients with OC in the United States, as the SEER program only covers nearly half of all cancer cases in the country.

## 5. Conclusions

The findings of this large study of adults with OC strongly suggest a sex difference in CVD mortality in this population, with males having a higher risk than female adults. This sex difference was more prominent among young and middle-aged adults. Our findings highlight the need to increase awareness, screening, and implementation of optimal cardiovascular health after diagnosis of OC, especially among young men, to enhance their survival and quality of life. With a large overlap of CVD risk factors and ocular malignancy, and cardiometabolic conditions often associated with mortality among patients with ocular malignancies, CVD risk screening in patients with OC, especially among young and middle-aged adults, is crucial due to the elevated risk of CVD mortality observed in the present study and the reported impaired quality of life associated with the diagnosis of OC in some patients. Thus, the integration of CVD screening in ocular cancer care has the potential to improve outcomes among this relatively small but significant patient population of OC survivors. Further evaluations of the role of radiation therapy on sex differences in the risk of CVD among patients with OC are warranted.

## Figures and Tables

**Table 1:** Characteristics of participants with ocular cancers according to sex, SEER, 2000–2021.

	Sex		*p* Value
Characteristics	Male (N = 6307)	Female (N = 5153)	
Age, years, %			0.012
18–44	794 (12.6)	705 (13.7)	
45–64	2755 (43.7)	2114 (41.0)	
≥65	2758 (43.7)	2334 (45.3)	
Year of diagnosis, %			0.610
2000–2004	1271 (20.2)	1020 (19.8)	
2005–2009	1298 (20.6)	1103 (21.4)	
2010–2014	1549 (24.6)	1227 (23.8)	
2015–2021	2189 (34.7)	1803 (35.0)	
Race and ethnicity, %			0.406
Non-Hispanic White	5383 (85.3)	4417 (85.7)	
Non-Hispanic Black	107 (1.7)	93 (1.8)	
Non-Hispanic Asian or Pacific Islander	163 (2.6)	117 (2.3)	
Hispanic	518 (8.2)	436 (8.5)	
Other ^1^	136 (2.2)	90 (1.7)	
Region, %			0.049
Midwest	382 (6.1)	322 (6.2)	
Northeast	902 (14.3)	829 (16.1)	
South	1437 (22.8)	1166 (22.6)	
West	3586 (56.9)	2836 (55.0)	
Marital status, married, %	4025 (63.8)	2602 (50.5)	<0.001
Median household income, %			0.507
<$75,000	3470 (55.0)	2867 (55.6)	
≥$75,000	2837 (45.0)	2286 (44.4)	
Location, rural, %	899 (14.3)	706 (13.7)	0.399
Primary site of tumor, %			<0.001
Choroid	3811 (60.4)	3431 (66.6)	
Ciliary body	513 (8.1)	531 (10.3)	
Conjunctiva	1019 (16.2)	520 (10.1)	
Orbit	305 (4.8)	190 (3.7)	
Other sites/unspecified	659 (10.4)	481 (9.3)	
Histology, %			<0.001
Uveal melanoma	4308 (68.3)	3949 (76.6)	
Conjunctival melanoma	286 (4.5)	263 (5.1)	
Melanoma, other sites	313 (5.0)	280 (5.4)	
Conjunctival squamous cell carcinoma	683 (10.8)	222 (4.3)	
Other histologic subtypes	717 (11.4)	439 (8.5)	
Tumor stage, %			0.686
Localized	4101 (65.0)	3373 (65.5)	
Regional	442 (7.0)	332 (6.4)	
Distant	177 (2.8)	147 (2.9)	
Unknown/unstaged	1586 (25.1)	1301 (25.2)	
Laterality, %			0.243 ^1^
Unilateral	6252 (99.1)	5097 (98.9)	
Bilateral	55 (0.9)	56 (1.1)	
Diagnostic confirmation, %			<0.001
Clinical diagnosis	335 (5.3)	364 (7.1)	
Direct visualization	736 (11.7)	706 (13.7)	
Other methods	417 (6.6)	391 (7.6)	
Positive histology	3839 (60.9)	2730 (53.0)	
Radiography	886 (14.0)	854 (16.6)	
Unknown	94 (1.5)	108 (2.1)	
Time from diagnosis to treatment, %			<0.001
<1 month	3280 (52.0)	2478 (48.1)	
1–2 months	1327 (21.0)	1276 (24.8)	
≥3 months	250 (4.0)	216 (4.2)	
Unknown	1450 (23.0)	1183 (23.0)	
Initial treatment, % ^2^			
Surgery	2873 (46.0)	1987 (38.8)	<0.001
Chemotherapy	329 (5.2)	208 (4.0)	0.003
Radiation	3403 (54.2)	3162 (61.7)	<0.001

Characteristics are presented as frequency (percentages). ^1^ Other race and ethnicity include American Indian/Alaska Native, and unknown or other race or ethnic groups. ^2^ Treatment groups are not mutually exclusive; thus, percentages may add up to more than 100.

**Table 2:** Sex difference (male vs. female) in mortality outcomes among patients with ocular cancer, SEER, 2000–2021 ^1^.

		Age-Adjusted	Multivariable Adjusted ^2^	Propensity Score Matched
Mortality outcomes	N	HR (95%CI)	HR (95%CI)	HR (95%CI)
All-cause	4561	1.14 (1.07–1.21)	1.20 (1.13–1.27)	1.17 (1.09–1.25)
Ocular cancer	946	0.99 (0.87–1.12)	1.02 (0.89–1.16)	1.01 (0.87–1.16)
Cardiovascular disease	694	1.41 (1.21–1.64)	1.54 (1.31–1.81)	1.52 (1.27–1.82)

CI: confidence intervals, HR: hazard ratio, N: number (frequency). ^1^ Reported hazard ratios are for males compared to females, with females being the referent group. ^2^ Adjusted for age at diagnosis, year of diagnosis, race and ethnicity, marital status, geographic region, location, income, histology, tumor site, tumor stage, laterality, mode of diagnosis, time from diagnosis to treatment, and initial cancer therapy.

**Table 3:** Characteristics of variables used for propensity score matching among patients with ocular cancers according to sex, SEER, 2000–2021.

	Sex		ASD	*p* Value
Characteristics	Male (N = 4490)	Female (N = 4490)		
Age, years, %				0.732
18–44	670 (14.9)	678 (15.1)		
45–64	1985 (44.2)	1948 (43.4)	0.009	
≥65	1835 (40.9)	1864 (41.5)	0.015	
Year of diagnosis, %				0.741
2000–2004	888 (19.8)	851 (19.0)		
2005–2009	941 (21.0)	967 (21.5)	0.006	
2010–2014	1083 (24.1)	1077 (24.0)	0.002	
2015–2021	1578 (35.1)	1595 (35.5)	0.011	
Race and ethnicity, %			0.013	0.557
White	3838 (85.5)	3812 (84.9)		
Non-white	652 (14.5)	678 (15.1)		
Region, %				0.301
Midwest	258 (5.7)	273 (6.1)	0.003	
Northeast	689 (15.3)	747 (16.6)	0.023	
South	998 (22.2)	991 (22.1)	0.002	
West	2545 (56.7)	2479 (55.2)		
Marital status, married, %	2625 (58.5)	2591 (57.7)	0.016	0.467
Median household income, %			0.013	0.687
<USD 75,000	2462 (54.8)	2481 (55.3)		
≥USD 75,000	2028 (45.2)	2009 (44.7)		
Location, rural, %	623 (13.9)	605 (13.5)		0.583
Histology, %				0.894
Uveal melanoma	3425 (76.3)	3428 (76.3)	0.003	
Conjunctival melanoma	226 (5.0)	222 (4.9)	0.003	
Melanoma, other sites	229 (5.1)	233 (5.2)	0.020	
Conjunctival squamous cell carcinoma	386 (8.6)	367 (8.2)	0.018	
Other histologic subtypes	224 (5.0)	240 (5.3)		
Tumor stage, %				0.870
Localized	2983 (66.4)	2982 (66.4)	0.003	
Regional	276 (6.1)	278 (6.2)	0.002	
Distant	108 (2.4)	120 (2.7)	0.003	
Unknown/unstaged	1123 (25.0)	1110 (24.7)		
Laterality, %			0.000	0.529
Unilateral	4447 (99.0)	4441 (98.9)		
Bilateral	43 (1.0)	49 (1.1)		
Diagnostic confirmation, %				0.335
Clinical diagnosis	308 (6.9)	350 (7.8)	0.028	
Direct visualization	617 (13.7)	627 (14.0)	0.000	
Other methods	338 (7.5)	332 (7.4)	0.003	
Positive histology	2402 (53.5)	2316 (51.6)	0.019	
Radiography	742 (16.5)	770 (17.1)	0.001	
Unknown	83 (1.8)	95 (2.1)		
Time from diagnosis to treatment, %				0.455
<1 month	2203 (49.1)	2155 (48.0)	0.015	
1–2 months	1084 (24.1)	1140 (25.4)	0.039	
≥3 months	177 (3.9)	190 (4.2)	0.006	
Unknown	1026 (22.9)	1005 (22.4)		
Initial treatment, % ^1^				
Surgery	1718 (38.6)	1690 (37.9)	0.004	0.467
Chemotherapy	180 (4.0)	187 (4.2)	0.011	0.709
Radiation	2780 (61.9)	2815 (62.7)	0.020	0.446

ASD: Absolute standardized difference. Characteristics are presented as frequency (percentages). ^1^ Treatment groups are not mutually exclusive; thus, percentages may add up to more than 100.

**Table 4:** Characteristics of participants with ocular cancers according to sex, TCR, 1995–2019.

	Sex		*p* Value
Characteristics	Male (N = 1501)	Female (N = 1331)	
Age, years, %			0.092
18–44	223 (14.9)	206 (15.5)	
45–64	634 (42.2)	509 (38.2)	
≥65	644 (42.9)	616 (46.3)	
Year of diagnosis, %			0.340
1995–1999	217 (14.5)	197 (14.8)	
2000–2004	272 (18.1)	226 (17.0)	
2005–2009	286 (19.1)	257 (19.3)	
2010–2014	269 (17.9)	275 (20.7)	
2015–2019	457 (30.4)	376 (28.2)	
Race and ethnicity, %			0.096
Non-Hispanic White	1279 (85.2)	1111 (83.5)	
Non-Hispanic Black	25 (1.7)	32 (2.4)	
Hispanic	180 (12.0)	160 (12.0)	
Other ^1^	17 (1.1)	28 (2.1)	
Health insurance			0.675
Not insured	49 (3.3)	45 (3.4)	
Medicaid	19 (1.3)	11 (0.8)	
Medicare	277 (18.5)	273 (20.5)	
Private	387 (25.8)	332 (24.9)	
Other	223 (14.9)	195 (14.7)	
Unknown	546 (36.4)	475 (35.7)	
Census tract poverty indicator, %			0.166
<10%	687 (45.8%)	619 (46.5%)	
10–20%	462 (30.8%)	436 (32.8%)	
>20%	301 (20.1%)	246 (18.5%)	
Unknown	51 (3.4%)	30 (2.3%)	
Location, rural, %	274 (18.3)	242 (18.2)	0.960
Primary site of tumor, %			0.004
Choroid	829 (55.2)	776 (58.3)	
Ciliary body	170 (11.3)	172 (12.9)	
Conjunctiva	175 (11.7)	101 (7.6)	
Other sites/unspecified	327 (20.4)	282 (21.2)	
Histology, %			0.004
Uveal melanoma	991 (66.0)	942 (70.8)	
Conjunctival melanoma	57 (3.8)	60 (4.5)	
Other histologic subtypes	453 (30.2)	329 (24.7)	
Tumor stage, %			0.046
Localized	883 (58.8)	747 (56.1)	
Regional	121 (8.1)	126 (9.5)	
Distant	78 (5.2)	49 (3.7)	
Unknown/unstaged	419 (27.9)	409 (30.7)	
Laterality, %			0.822
Unilateral	1447 (96.4)	1281 (96.2)	
Bilateral	54 (3.6)	50 (3.8)	
Initial treatment, % ^2^			
Surgery	670 (44.6)	500 (37.6)	<0.001
Radiation	380 (25.3)	380 (28.5)	0.127
Chemotherapy	65 (4.3)	55 (4.1)	0.550

Characteristics are presented as frequency (percentages). ^1^ Other race and ethnicity include Non-Hispanic Asian or Pacific Islander, American Indian/Alaska Native, and unknown or other race or ethnic groups. ^2^ Treatment groups are not mutually exclusive; thus, percentages may add up to more than 100.

**Table 5:** Sex difference (male vs. female) in mortality outcomes among patients with ocular cancer, TCR, 1995–2019 ^1^.

		Age-Adjusted	Multivariable Adjusted ^2^
Mortality outcomes	N	HR (95%CI)	HR (95%CI)
All-cause	1302	1.20 (1.07–1.33)	1.14 (1.02–1.28)
Ocular cancer	712	1.03 (0.89–1.19)	1.03 (0.88–1.19)
Cardiovascular disease	242	1.45 (1.13–1.88)	1.34 (1.03–1.76)

CI: confidence intervals, HR: hazard ratio, N: number (frequency). ^1^ Reported hazard ratios are for males compared to females, with females being the referent group. ^2^ Adjusted for age at diagnosis, year of diagnosis, race and ethnicity, location, income, census tract poverty, health insurance status, histology, tumor site, tumor stage, laterality, and initial cancer therapy.

## Data Availability

Data used for this study are publicly available from the National Cancer Institute at https://seer.cancer.gov/ (accessed on 18 June 2024).

## Abbreviations

The following abbreviations are used in this manuscript:

ASD	Absolute standardized difference
CI	Confidence intervals
CVD	Cardiovascular disease
HR	Hazard ratio
ICD	International classification of diseases
OC	Ocular cancer
SEER	Surveillance, epidemiology, and end results
